# V-ATPase in cancer progression: Two sides of the same coin

**DOI:** 10.18632/oncotarget.26218

**Published:** 2018-10-16

**Authors:** Mohd Saqib, Bibhuti B. Mishra

**Affiliations:** Bibhuti B. Mishra: Albany Medical College, Albany, New York, USA

**Keywords:** ATPase, tumor microenvironment, metastasis, cytotoxic T-cells, hematopoietic cells

Vacuolar ATPase (V-ATPase) is an ATP driven proton pump that is present on the membrane of intracellular vesicles like endosomes, lysosomes, where it is responsible for maintaining intracellular and vesicular pH under normal physiologic conditions. However, it is also expressed on the plasma membrane of cancer cells where it acidifies the extracellular environment, thus promoting growth and metastasis of cancer cells. The structure and function of V-ATPase are highly conserved across species. Structurally the V-ATPase has two domains: the membrane-bound “V_0_” domain and the cytoplasmic “V_1_” domain [1]. Some of the V-ATPase subunits have multiple isoforms and their expression is tissue specific. For example, the “a” subunit that spans both V_0_ and V_1_ domains, has 4 isoforms and the “a2 isoform of V-ATPase” (a2V), is expressed on cells of the hematopoietic origin and endothelial cells [2]. Tumor-derived a2V plays a pathogenic role in the progression and metastasis of solid tumors. Tumor cells produce a2V derived peptides that serve as potent chemo attractants for tumor associated neutrophils that subsequently produce growth factors and inflammatory eicosanoids to aid in metastasis of solid tumors [3, 4]. Although the role of tumor derived a2V as a tumor promoting factor is well established in various studies [5], [6] the role of immune cell-derived a2V in cancer progression has not been explored, given its expression by a number of innate and adaptive immune cells of hematopoietic origin.

In this work, Sahoo et al. examined the role of hematopoietic cell derived a2V in breast cancer growth and metastasis using a mouse model of breast tumor progression [7]. Previous studies have demonstrated that selective deletion of a2V in cancer cells blunts the cancer growth leading to the improved outcome. But, none of the published studies ever addressed the role of a2V in immune cells that normally infiltrate the tumor microenvironment (TME), and affects the outcome of tumor progression. Sahoo et al. took the Cre-LoxP based approach of generating a conditional deletion of a2V in hematopoietic stem cells (HSC) and demonstrated that a2V in immune cells, unlike the cancer cells, possesses an anti-tumor function. a2V conditional knock-out (KO) mice developed faster growing and more invasive breast cancer than the control littermates upon implantation of breast tumor in the mammary fat pad. This is a very interesting finding and opens many questions. To begin with, they analyzed the TME for the presence of infiltrating leukocytes by flow cytometry and cytokines/chemokines likely to contribute to the inflammation at the tumor site by targeted RNAseq. Interestingly, they discovered that the number of infiltrating CD4^+^ T-helper (Th) and CD8^+^ cytotoxic T cells (Tc) were significantly reduced, while the numbers of immune suppressive myeloid-derived suppressor cells (MDSC) were significantly increased in the TME of a2V KO mice. The TME of the a2V-KO mice is conducive for tumor growth by offering an anti-inflammatory and anti-apoptotic milieu. In contrast, they attribute the smaller size of the tumor in control mice to a pro-inflammatory TME with a higher number of CD4^+^ Th and CD8^+^ Tc cells.

To decipher if the reduction of infiltrating CD4^+^ Th and CD8^+^ Tc cells is due to a defect in the inability to migrate or a defect in their production, they analyzed various T cell subsets in the periphery (blood and spleen), site of maturation (thymus) and site of generation (bone marrow). They discovered that lack of a2V in HSCs causes a defect in production and maturation of both CD4^+^ Th and CD8^+^ Tc cells in bone marrow and thymus, respectively. Therefore, the abundance of both the CD4^+^ Th and CD8^+^ Tc cells is less in circulation and TME of a2V KO mice. Although the authors do not present any evidence of this defect mechanistically, they speculate that this defect in production and maturation of both CD4^+^ Th and CD8^+^ Tc cells in a2V conditional knock-out mice might be due (i) a defect in glycosylation and/or (ii) cell death pathways. This speculation is based on previously published reports that deletion of a2V relocates glycosyltransferase enzyme to endosomes and leads to enhanced cell death by inducing autophagy. Sahoo et al. went one step further to delineate which cells of the αβ T cell populations are important to control tumor growth and metastasis. For this, they depleted either the CD4^+^ Th or the CD8^+^ Tc cells from normal mouse and monitored tumor growth and metastasis of implanted breast tumor cells. They demonstrate that lack of CD8^+^ Tc cells from the periphery results in larger, faster growing and metastatic breast tumor. The findings emerged from this study offers a new rationale for exploring the role of a2V in cancer biology and immunology in general. The work of Sahoo et al. raised a few important questions that deserve attention. The inherent lymphopenia observed in naïve a2V KO mice suggests that a2V might be playing a role in lymphopoiesis. Does a2V regulate T-cell numbers by controlling a crucial lineage commitment step of the hematopoietic progenitors, or does it affect T-cell survival by regulating cell death pathways? Does a2V deficiency in HSCs compromise lymphoid cell generation at the expense of dysregulated myelopoiesis, as the metastasizing breast cancers are infiltrated with MDSC-like cells? Future experiments to address these questions may shed light on the mechanisms of antitumor effects of immune cell-derived a2V especially in maintaining a T-cell pool in the tumor-bearing host.
